# Impact of age on efficacy of postoperative oxaliplatin-based chemotherapy in patients with rectal cancer after neoadjuvant chemoradiotherapy

**DOI:** 10.18632/oncotarget.7544

**Published:** 2016-02-21

**Authors:** Xuan-zhang Huang, Peng Gao, Yong-xi Song, Jing-xu Sun, Xiao-wan Chen, Jun-hua Zhao, Bin Ma, Jun Wang, Zhen-ning Wang

**Affiliations:** ^1^ Department of Chemotherapy and Radiotherapy, the Second Affiliated Hospital and Yuying Children's Hospital of Wenzhou Medical University, Lucheng District, Wenzhou City 325027, P.R. China; ^2^ Department of Surgical Oncology and General Surgery, the First Hospital of China Medical University, Heping District, Shenyang City 110001, P.R. China

**Keywords:** rectal cancer, adjuvant chemotherapy, age, elderly, SEER program

## Abstract

**Background:**

Clinical practice guidelines focusing on age-related adjuvant chemotherapy for rectal cancer are currently limited. The present study aimed to explore the impact of age on the efficacy of adjuvant oxaliplatin-based chemotherapy in patients with rectal cancer after neoadjuvant chemoradiotherapy.

**Methods:**

We performed a retrospective cohort analysis using data from the Surveillance, Epidemiology, and End Results-Medicare-linked database from 1992–2009. We enrolled patients with yp stages I–III rectal cancer who received neoadjuvant chemoradiotherapy and underwent curative resection. The age-related survival benefit of adding oxaliplatin to adjuvant 5-fluorouracil (5-FU) chemotherapy was evaluated using Kaplan–Meier survival analysis with propensity score-matching and Cox proportional hazards models.

**Results:**

Comparing the oxaliplatin group with the 5-FU group, there were significant interactions between age and chemotherapy efficacy in terms of overall survival (OS) (*p* for interaction = 0.017) among patients with positive lymph nodes (ypN+). Adding oxaliplatin to 5-FU could prolong survival in patients aged < 73 years and ypN+ category, and but did not translate into survival benefits in patients aged ≥ 73 years and ypN+ category. No significant interactions were observed among ypN− patients, and oxaliplatin did not significantly improve OS, regardless of age.

**Conclusions:**

In patients with rectal cancer who have already received neoadjuvant chemoradiotherapy and undergone curative resection, adding oxaliplatin to 5-FU could prolong OS in patients aged < 73 years and ypN+ category. However, adding oxaliplatin did not translate into survival benefits in patients age ≥ 73 years and ypN+ category, or in ypN− patients.

## INTRODUCTION

Rectal cancer is one of the most common diagnosed cancers worldwide, with high morbidity [1]. The introduction of multidisciplinary treatments with adjuvant chemoradiotherapy and total mesorectal excision has contributed to improved survival benefits in patients with rectal cancer [2–4]. However, its high mortality and poor prognosis, mainly associated with high rates of recurrence and metastasis, remain challenges for both patients and oncologists [5, 6]. Preoperative neoadjuvant chemoradiotherapy is regarded as the standard treatment for locally-advanced rectal cancer. The National Comprehensive Cancer Network (NCCN) Clinical Practice Guidelines currently recommend adjuvant 5-fluorourcil (5-FU)-based chemotherapy for the treatment of rectal cancer with positive lymph nodes (ypN+) [7].

Although several studies have evaluated the impact of age on the efficacy of adjuvant chemotherapy in patients with rectal cancer, it remains debatable if treatment recommendations for adjuvant chemotherapy should consider age as a factor [8–10]. A lack of high-level evidence means that there is currently no uniform consensus regarding the impact of age on the efficacy of adjuvant chemotherapy. The latest NCCN Guidelines for Older Adult Oncology take account of age in terms of adjuvant chemotherapy in rectal cancer patients, but the age-related cut-off points have not been validated [11]. Furthermore, the effect of age on adjuvant chemotherapy in elderly patients is usually extrapolated from younger patients, because clinical guidelines tend to be based on studies from which elderly patients were often excluded [12, 13].

Clinically, age is an important factor affecting the choice of adjuvant chemotherapy in patients with rectal cancer, because elderly patients are more likely to have high comorbidity and poor performance status, which may increase treatment-related complications or death, thus outweighing the survival benefits [14, 15]. Indeed, studies have reported that the life expectancy of rectal cancer patients is related to age and concomitant comorbidities [16–18]. Age-related decisions about the use of adjuvant chemotherapy in patients with rectal cancer are thus a difficult and complex process, considering the balance between the likely risks and survival benefits.

The purpose of the present study was to explore the impact of age on the efficacy of adjuvant oxaliplatin-based chemotherapy in patients with rectal cancer who had already received neoadjuvant chemoradiotherapy.

## RESULTS

### Patient characteristics

Our study comprised 763 patients with rectal cancer who received neoadjuvant chemoradiotherapy recommended by the NCCN. The detailed baseline characteristics of patients are presented in Table 1. Details of race, marital status, median household income, level of education, histologic type, and intestinal perforation were not presented in Table 1, because the SEER-Medicare rules require that cell sizes less than eleven in a table must be suppressed.

**Table 1:** Clinicopathologic features of patients with different chemotherapy regimens

	5–FU	Oxaliplatin	*P* value
Gender			0.858
Male	321	131	
Female	219	92	
Age at diagnosis, years			0.001
66–70	184	105	
71–75	175	70	
> 75	181	48	
Residence location			0.025
Big Metro	261	127	
Metro or Urban	199	77	
Less Urban or Rural	80	19	
Year of diagnosis			< 0.001
1992–2000	125	0	
2001–2004	251	26	
2005–2008	164	197	
Histologic grade			0.980
Well	35	14	
Moderate	360	146	
Poor/Undifferentiated	87	37	
Unknown	58	26	
ypT category			0.398
ypT1–2	146	50	
ypT3	367	160	
ypT4	27	13	
ypN category			0.795
ypN0	339	134	
ypN1a	85	34	
ypN1b	59	26	
ypN2a	36	16	
ypN2b	21	13	
ypTNM stage			0.315
ypTNM I	113	36	
ypTNM II	226	98	
ypTNM III	201	89	
Intestinal obstruction			0.834
No	484	201	
Yes	56	22	
HCC risk score			0.503
1st quartile	143	56	
2nd quartile	131	45	
3rd quartile	152	67	
4th quartile	114	55	
Number of examined lymph node			0.007
≥ 12	200	106	
< 12	340	117	
Postoperative radiotherapy			0.001
No	455	208	
Yes	85	15	

**Abbreviation:** HCC, Hierarchical Condition Categories; No-chemo, without postoperative chemotherapy; 5-FU, 5-fluorouracil.

### Overall comparison of oxaliplatin and 5-FU groups

According to univariate analysis, the addition of oxaliplatin did not significantly improve OS (hazard ratio [HR] = 0.883, 95% confidence interval [CI] = 0.634–1.230, *p* = 0.462) compared with 5-FU. We modeled the interaction between age and the efficacy of oxaliplatin using age as a continuous variable and found no significant interaction in terms of OS (*p* = 0.719). Compared with patients in the 5-FU group, patients in the oxaliplatin group experienced significantly higher rates of most adverse events including neutropenia (30.04% vs 8.52, Δ% = 21.52%, *p* < 0.001), nausea or vomiting (30.04% vs 13.33, Δ% = 16.71% *p* < 0.001), dehydration (18.39% vs 10.19, Δ% = 8.20%, *p* = 0.002), and thrombocytopenia (Δ% = 7.49%, *p* < 0.001). Furthermore, elderly patients who received oxaliplatin had significantly higher incidence of acute renal failure compared with younger patients (Δ% = 7.27%, *p* = 0.010), while elderly patients had slightly higher incidences of infection (Δ% = 4.17%), nausea or vomiting (Δ% = 1.22%), dehydration (Δ% = 4.21%), diarrhea (Δ%=1.14%), thrombocytopenia (Δ% = 3.41%), ischemic heart disease (Δ% = 3.64%), congestive heart failure (Δ% = 1.78%), and cardiac dysrhythmia (Δ% = 3.22%), though the differences were not significant. The detailed chemotherapy-related adverse events are summarized in Supplementary Table 1.

### Comparison of ypN+ oxaliplatin and 5-FU groups

**Figure 1:**
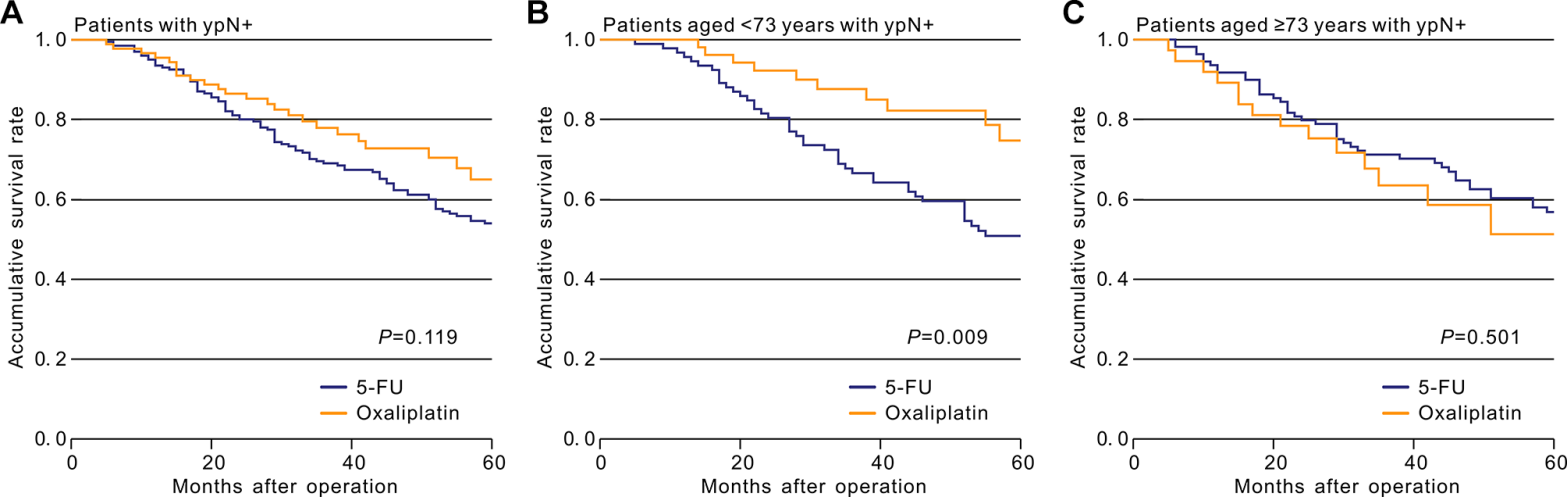
Kaplan-Meier comparison of overall survival among ypN+ patients who received different postoperative treatment (**A**) All patients with ypN+; (**B**) Patients aged < 73; (**C**) Patients aged ≥ 73.

We also determined the impact of age on the efficacy of chemotherapy among patients with ypN+ category. In univariate analysis, the addition of oxaliplatin did not significantly improve OS (HR = 0.697, 95% CI = 0.443–1.097, *p* = 0.119; Figure 1A) compared with the 5-FU group. Modeling age as a continuous variable, there was a marginally significant interaction between age and the efficacy of oxaliplatin in terms of OS (*p* for interaction = 0.082).

**Figure 2:**
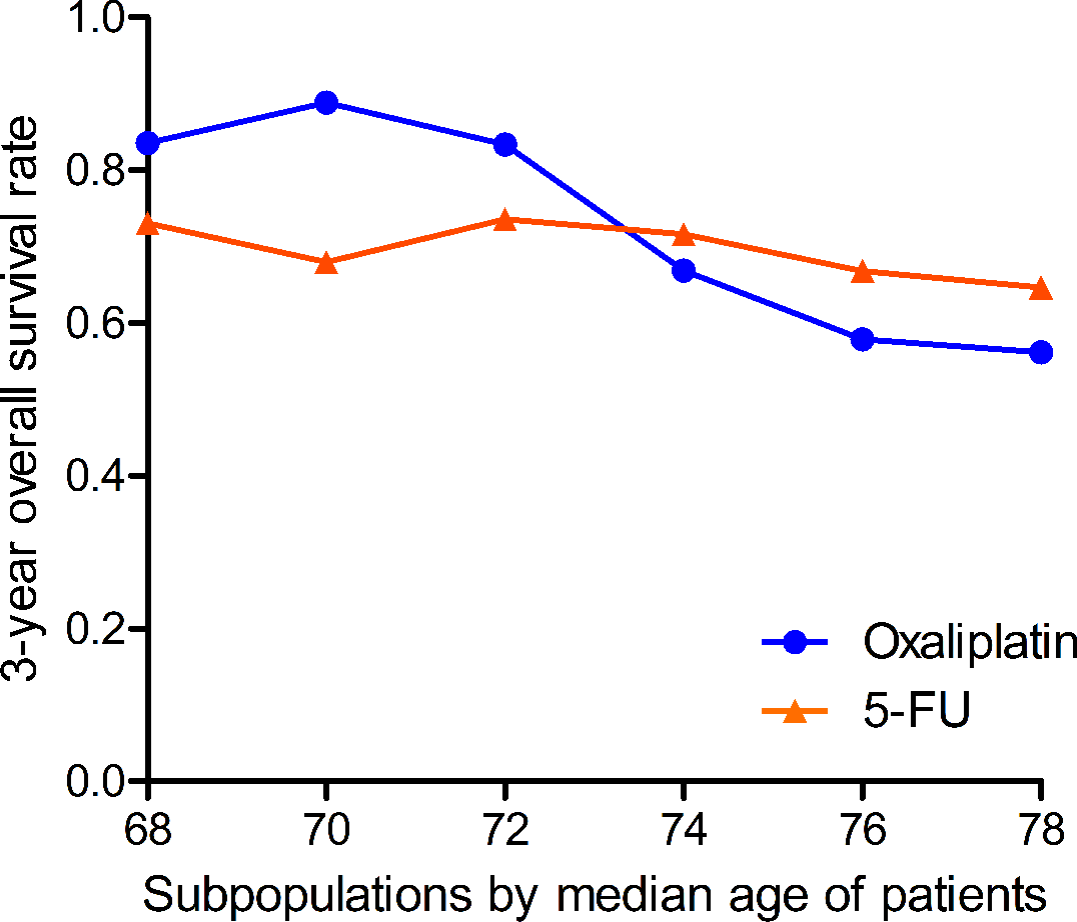
Identification of the impact of age on the efficacy of chemotherapy in terms of overall survival

The results of STEPP analysis showed that the efficacy of chemotherapy in terms of OS was reversed at age 73 years; the addition of oxaliplatin improved OS compared with 5-FU alone in patients younger than 73 years, but there was no obvious benefit among patients aged ≥ 73 years (Figure 2). Modeling age as a dichotomous variable (age ≥ 73 years and < 73 years), there was a significant interaction between age and efficacy of chemotherapy in terms of OS (*p* for interaction = 0.017).

**Figure 3:**
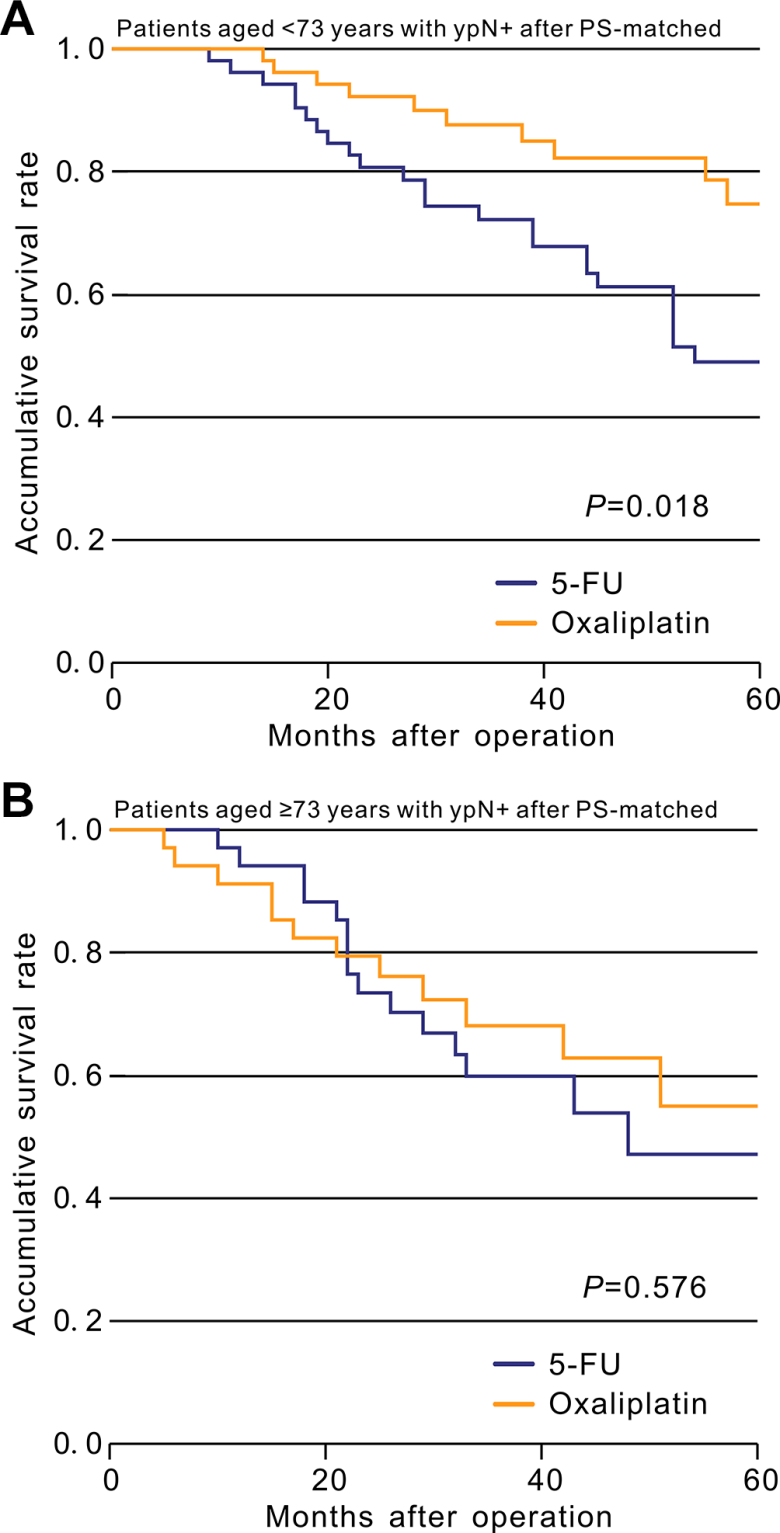
After PS-matched, Kaplan-Meier comparison of overall survival between patients in the 5-FU group and in the oxaliplatin group stratified by age (**A**) Patients aged < 73; (**B**) Patients aged ≥ 73.

Kaplan–Meier unadjusted survival analysis stratified by age (< 73 and ≥ 73 years) was used to compare survival between the oxaliplatin and 5-FU groups and produced results consistent with those of the STEPP method. Oxaliplatin significantly improved OS in patients younger than 73 years (HR = 0.411, 95% CI = 0.206–0.818, *p* for log-rank = 0.009, Figure 1B), but not in those aged ≥ 73 years (HR = 1.229, 95% CI = 0.670–2.255, *p* for log-rank = 0.501, Figure 1C), compared with the 5-FU group. Moreover, PS-matched cohorts based on related variables were generated for survival analysis, and the difference in OS between the two treatment groups among patients aged < 73 years remained significant (HR = 0.409, 95% CI = 0.196–0.856, *p* = 0.018, Figure 3A), while the difference among patients aged ≥ 73 years remained insignificant (HR = 0.805, 95% CI = 0.376–1.721, *p* = 0.576, Figure 3B).

Cox proportional hazards models were performed, including all the covariates related to survival. The oxaliplatin group had significantly better OS (HR = 0.449, 95% CI = 0.225–0.899, *p* = 0.024, Table 2) than the 5-FU group among patients aged < 73 years, but the difference in OS between the oxaliplatin group and 5-FU group among patients aged ≥ 73 years was not significant (HR = 1.484, 95% CI = 0.701–3.142, *p* = 0.303, Table 2).

**Table 2:** Cox proportional hazards model for ypN+ patients stratified by age

	HR	95%CI	*P*
Age < 73 years			
Histologic grade			
Well	1.316	0.315–5.503	0.707
Moderate	1		
Poor/Undifferentiated	2.395	1.336–4.295	0.003
Unknown	0.565	0.134–2.383	0.437
Chemotherapy regimens			
5-FU	1		
Oxaliplatin	0.449	0.225–0.899	0.024
Age ≥ 73 years			
ypN category			
ypN1a	1		
ypN1b	0.951	0.444–2.036	0.898
ypN2a	1.818	0.940–3.516	0.076
ypN2b	3.217	1.484–6.974	0.003
Year of diagnosis			
1992–1996	39.044	4.084–373.260	0.001
1997–2000	2.237	0.944–5.304	0.068
2001–2004	.920	0.451–1.878	0.820
2005–2008	1		
HCC risk score			
1st quartile	1		
2nd quartile	0.661	0.303–1.441	0.298
3rd quartile	0.780	0.347–1.756	0.548
4th quartile	2.361	1.206–4.624	0.012
Chemotherapy regimens			
5-FU	1		
Oxaliplatin	1.484	0.701–3.142	0.303

**Abbreviation:** 5-FU, 5-fluorouracil; CI, Confidential intervals; HCC, Hierarchical Condition Categories.

In terms of adverse events, the results in ypN+ patients in relation to age were similar to the results for the entire study population.

### Comparison of ypN− oxaliplatin and 5-FU groups

Univariate analysis of patients with ypN− rectal cancer showed that the addition of oxaliplatin did not significantly improve OS (HR = 1.061, 95% CI = 0.654–1.723, *p* = 0.811, Supplementary Figure 1) compared with 5-FU alone. Modeling age as a continuous variable, there was no significant interaction between age and efficacy of oxaliplatin in terms of OS (*p* for interaction = 0.238). The results regarding the effects of age on adverse events were similar in ypN− patients to those for the study population as a whole.

## DISCUSSION

Patients diagnosed with locally-advanced rectal cancer who have already received preoperative neoadjuvant chemoradiotherapy are recommended for postoperative chemotherapy. However, there is no consensus on whether adding oxaliplatin to 5-FU can improve their prognosis. Hong et al. completed a randomized controlled trial of ADjuvant Oxaliplatin in REctal cancer (ADORE) and demonstrated that postoperative adjuvant FOLFOX improved disease-free survival compared with fluorouracil plus leucovorin in patients with locally-advanced rectal cancer after preoperative chemoradiotherapy and total mesorectal excision [19]. In contrast, however, Nimeiri et al. performed an updated survival analysis of the randomized ECOG E3201 trial and found no difference in 5-year OS between patients who received 5-FU alone and those who received oxaliplatin-based postoperative chemotherapy [20]. Moreover, our previous analysis of data from the SEER–Medicare database demonstrated that adding oxaliplatin to 5-FU postoperative chemotherapy did not improve cancer-specific survival in patients who received neoadjuvant chemoradiotherapy [21]. However, these previous studies of the efficacy of postoperative chemotherapy in rectal cancer did not consider the impact of age.

The Adjuvant Colon Cancer END-points (ACCENT) group explored the impact of age on efficacy of oxaliplatin-based chemotherapy among patients with colon cancer and proposed that adding oxaliplatin to 5-FU could improve disease-free survival in patients aged 50–65 years, while patients aged ≥ 70 years experienced less benefit from the addition of oxaliplatin [22]. The FOWARC study evaluated preoperative FOLFOX concurrent with radiotherapy and showed higher pathological complete response and down-staging rates than other relevant studies [23]. Meanwhile, FOWARC notably included relatively younger patients (median age, 52 years) than other studies [4, 23–25]. Similarly, elderly patients with rectal cancer are known to be a highly heterogeneous subpopulation for adjuvant chemotherapy, with a high incidence of comorbidities, and many studies have therefore excluded this subpopulation, or it has only comprised a small percent of the overall study population. Indeed, the ADORE trial included patients with a median age of 55 years, and only 55 (17%) patients were aged ≥ 65 years, making it inappropriate to extrapolate the conclusions to elderly patients [19]. In contrast, our previous study analyzed patients aged ≥ 66 years from the SEER–Medicare database [21]. It is therefore possible that the apparent discrepancies between the results may be attributable to differences in patient age, and that the efficacy of chemotherapy may be age-dependent.

To the best of our knowledge, the present study was the first to explore the effect of age on the efficacy of adjuvant oxaliplatin-based chemotherapy. Although the differences did not reach statistical significance in a crude analysis of data for rectal cancer patients, our results nevertheless indicated that the efficacy of adding oxaliplatin to 5-FU was reversed at a specific age point. Several previous studies have reported that postoperative pathologic stage could accurately estimate prognosis and thus play an important role in clinical decision making in terms of the use and efficacy of postoperative chemotherapy [26–28]; however, postoperative chemotherapy is recommended by the NCCN Clinical Practice Guidelines regardless of the postoperative pathological results [7]. We therefore conducted a detailed analysis stratified by ypTNM stage (ypN+ and ypN−) to explore the relationship between chemotherapy efficacy and age using the STEPP method. Tests for interaction showed that patient age could play an important role in predicting the efficacy of chemotherapy, and indicated that, compared with 5-FU alone, adding oxaliplatin to 5-FU could prolong survival in ypN+ patients aged < 73 years, but did not translate into significant survival benefits in ypN+ patients aged ≥ 73 (Figure 2, 3), in accord with the results of a study evaluating age and chemotherapy in colon cancer [22]. However, oxaliplatin did not significantly improve survival in ypN− patients regardless of age, and a test for interaction showed that age had no effect on the lack of survival benefits. The stability of our results was confirmed by PS-matched analysis (Figure 3) and Cox proportional hazards model (Table 2).

Several previous studies reported that the use of adjuvant chemotherapy would decrease with increasing patient age [29, 30], mainly because clinicians considered that the high comorbidity and poor performance status in older patients would have a negative influence on survival [31]. However, although several studies have demonstrated that patient age may be an important factor for predicting prognosis in patients with rectal cancer, no specific studies have evaluated the impact of age on the efficacy of adjuvant chemotherapy in rectal cancer patients who have already received preoperative neoadjuvant chemoradiotherapy. Moreover, the NCCN Clinical Practice Guidelines for Older Adult Oncology provide no detailed evidence or clinical decision-making strategies regarding postoperative chemotherapy for elderly patients with rectal cancer [11]. Further evidence is therefore needed to validate the decreased use of adjuvant chemotherapy in elderly patients. Furthermore, there is no consistent definition of elderly age for patients with rectal cancer. The results of the current study suggest that the selection of adjuvant chemotherapy should be based on patient age, and STEPP analysis indicated a cut-off of age for oxaliplatin-based chemotherapy of 73 years, as confirmed by Cox proportional hazards model analysis. In addition, however, the impact of age on adjuvant chemotherapy differed according to postoperative pathologic stage, with patient age impacting on the efficacy of adjuvant chemotherapy in ypN+, but not ypN− patients. It is possible that a survival benefit was difficult to detect among ypN− patients treated with adjuvant chemotherapy because of their better rate of survival without chemotherapy, making it harder to detect an improvement [8]. We also found that ypN+ patients aged ≥ 73 years in the 4th quartile of HCC risk had poorer prognoses than those in the 1st quartile, indicating that a high HCC risk score was a risk factor. These results suggest that decisions regarding adjuvant chemotherapy should not be made on the basis of patient age alone, and further studies are urgently needed to explore the need for careful assessment of performance status, comorbidities, treatment risks, and life expectancy in the selection of optimal treatment modalities.

Chemotherapy-related adverse events are a concern in clinical practice. Our study showed that adding oxaliplatin to 5-FU could increase the incidence of adverse effects compared with 5-FU alone in terms of neutropenia, nausea or vomiting, dehydration, and thrombocytopenia. Adverse events were also more common in elderly patients who received oxaliplatin compared with younger patients (i.e., acute renal failure, infection, nausea or vomiting, dehydration, diarrhea, thrombocytopenia, ischemic heart disease, congestive heart failure, and cardiac dysrhythmia), indicating that the incidence of adverse events was age related, consistent with other studies [32, 33]. Age-related adverse events may thus mask or impact on the efficacy of oxaliplatin regimens in elderly subpopulations. Further large-scale, high-quality studies are needed to evaluate the impact of age on adverse events, and to improve patient selection for individual chemotherapy.

Several limitations of the present study should be addressed. First, this was a retrospective study, and despite the use of both PS-matched analysis and Cox proportional hazards model to account for known relevant confounding factors, we could not eliminate the possibility of other potential confounding factors that might have affected the use and efficacy of chemotherapy. Second, the data on adjuvant chemotherapy was from fee-for-service insurance using a “one-claim” algorithm, which may have influenced the representative nature of the included patients, resulting in heterogeneity [34, 35]. Third, our study could not determine how aggressively to treat patients such that the survival benefits from chemotherapy would outweigh the costs and risks. Furthermore, clinical treatment patterns may differ between younger and more elderly patients, and these differences may limit the applicability of the results to the overall rectal cancer population. Future studies are needed to determine the subpopulations of younger and more elderly patients who may derive survival benefit from adjuvant chemotherapy.

In conclusion, the addition of oxaliplatin to 5-FU chemotherapy could prolong OS in rectal cancer patients with prior neoadjuvant chemoradiotherapy and curative resection aged < 73 years with ypN+ status, but not in ypN+ patients aged ≥ 73 years, or in ypN− patients, irrespective of age.

## MATERIALS AND METHODS

### Ethics statement

The Permission to access the research data file in Surveillance, Epidemiology, and End Results (SEER)–Medicare program was obtained by the authors and (reference no. D6-MEDIC-821). All data were masked and no protected health information could be linked to individual patients. The study was approved by the Institutional Review Board of the First Hospital of China Medical University (reference no. [2012] 96).

### Data source

This study utilized data from the Surveillance, Epidemiology, and End Results (SEER)–Medicare database, based on collaboration among the SEER registries, the National Cancer Institute, and the Centers for Medicare and Medicaid Services, and which provides patient data through linkage of population-based SEER and Medicare claims data. The SEER program contains information on cancer patient demographics, tumor characteristics, cancer-related treatments, and cause of death, and includes approximately 28% of the US population [36]. Part A of Medicare comprises health-insurance data for approximately 97% of the US population aged ≥ 65 years, covering hospitals, skilled-nursing facilities, hospices, and home health care, while Part B comprises approximately 96% of beneficiaries, covering physician and outpatient services [37, 38].

### Eligible patient selection

The inclusion criteria for eligible patients were as follows: (1) aged ≥ 66 years who were diagnosed with primary rectal (SEER cancer site codes: 19.9 and 20.9) adenocarcinoma (SEER histology codes: 8000–8152, 8154–8231, 8243–8245, 8250–8576, 8940–8950, and 8980–8981) from 1992–2008; (2) underwent primary tumor resection with curative intent within 180 days of diagnosis; (3) received preoperative neoadjuvant therapy involving radiotherapy plus 5-FU or capecitabine recommended by NCCN; (4) received postoperative chemotherapy within 120 days of operation (5-FU group included all patients who received 5-FU or capecitabine alone, the oxaliplatin group included all patients who received 5-FU and leucovorin plus oxaliplatin or capecitabine plus oxaliplatin within 30 days of their first chemotherapy dose). The detailed drug codes based on National Drug Code and Health Care Financing Administration Common Procedure Coding System have been reported previously [21].

The exclusion criteria for patients were: (1) diagnosed with *in situ* tumor (yp stage 0: Tis N0 M0) because of the small sample size; (2) history of prior non-rectal cancer or diagnosis of non-rectal cancer 1 year after rectal cancer diagnosis; (3) received other postoperative chemotherapy regimens; (4) died during the immediate postoperative period (within 30 days); (5) incomplete pathological stage entries or diagnostic data; (6) diagnosed with pTxNxMx, because of inaccuracy of the data in the SEER–Medicare database; and (7) membership of a Medicare-sponsored health maintenance organization or lack of enrollment in Medicare Parts A and B from 12 months before through to 9 months after diagnosis.

### Study variables

Patient demographic characteristics including age and year at diagnosis, sex, race, geographic region, marital status, and socioeconomic status (household income and education level) were obtained from the SEER patient entitlement and diagnosis summary file. Socioeconomic status was used to summarize the median household income at census tract level and the percentage of people aged ≥ 25 years with < 12 years of education.

Disease characteristics including tumor stage, tumor grade, histological type, and comorbidity were analyzed. Postoperative pathological stage (ypTNM) was identified based on the seventh edition of the Union for International Cancer Control or American Joint Committee on Cancer tumor-node-metastasis (TNM) staging system [39, 40]. To control for the effects of comorbidities, analyses were adjusted according to the Centers for Medicare and Medicaid Services Hierarchical Condition Category (HCC) based on Medicare inpatient and outpatient claims for various comorbidities within the 12 months before rectal cancer diagnosis, where the HCC risk score summarizes the health care problems and predicts the future health care cost of a population compared with the average Medicare beneficiary (HCC = 1.0) [41]. In the current study, HCC risk score, calculated based on patient demographics and diagnostic profiles using software provided by the Centers for Medicare and Medicaid Services, was used to reflect the health conditions of the patients [42]. We also identified chemotherapy-related adverse events by assessing discharge diagnoses within 6 months of chemotherapy initiation. To avoid the effects of prediagnosis disease conditions on chemotherapy-related adverse events, disease conditions occurring in the 12 months before cancer diagnosis were not identified as adverse events. International Classification of Diseases, 9th Revision, Clinical Modification (ICD-9-CM) diagnostic codes was used to classify chemotherapy-related adverse events.

### Statistical analysis

Categorical variables were compared using χ^2^ test. Kaplan–Meier survival analysis and log-rank tests were used to compare overall survival (OS) between different chemotherapy groups. Subpopulation treatment effect pattern plot (STEPP) analysis was used to examine the relationship between treatment effect and the covariate of interest (i.e., age) and to validate the age-related cut-off points for treatment effects by estimating the treatment effects for a sequence of subpopulations, where the subpopulations corresponded to age values [43–45]. Multivariate analyses were performed using Cox proportional hazards models including all significant survival-related variables in univariate analysis.

In clinical practice, there were several significant differences between patients treated with or without chemotherapy, particularly in terms of comorbidities and age. We therefore used propensity score (PS)-matched analysis to deal with the selection bias caused by nonrandom assignment and to compare treatment effects [46–48]. Each patient's probability of receiving oxaliplatin or 5-FU depending on the covariates in each group was estimated by logistic regression models. Unexposed PS-matched cohorts with a balance of covariates and equivalent mean PS across treatment groups were generated and log-rank tests were applied to these PS-matched cohorts.

All statistical analyses were performed using R, version 3.1.1 (R Foundation for Statistical Computing, Vienna, Austria), STATA version 12.0 (Stata Corporation, College Station, TX, USA), SAS version 9.3 (SAS Institute, Cary, NC, USA), and PASW Statistics version 18.0 (SPSS, Inc., Somers, NY, USA). A two-sided *p* value < 0.05 was considered statistically significant for all analyses.

## SUPPLEMENTARY MATERIALS FIGURE AND TABLE



## Abbreviations

ACCENT: Adjuvant Colon Cancer End-Points
ADORE: ADjuvant Oxaliplatin in REctal cancer
CIs: confidence intervals
HCC: Hierarchical Condition Category
HR: hazard ratio
ICD-9-CM: International Classification of Diseases, 9th Revision, Clinical Modification
NCCN: National Comprehensive Cancer Network
OS: overall survival
PS: propensity score
SEER: Surveillance, Epidemiology, and End Results
STEPP: Subpopulation treatment effect pattern plot
TNM: tumor-node-metastasis
ypN+: positive lymph nodes
5-FU: 5-fluorourcil.
