# Supplementary material for: Impact of age on efficacy of postoperative oxaliplatin-based chemotherapy in patients with rectal cancer after neoadjuvant chemoradiotherapy
Source: Oncotarget. 2016 Feb 21;7(15):19643–53. doi: 10.18632/oncotarget.7544 (PMC4991408; doi:10.18632/oncotarget.7544)
Supplement: Supplementary file 1 [file oncotarget-07-19643-s001.pdf]

Impact of age on efficacy of postoperative oxaliplatin-based chemotherapy in patients with rectal cancer after neoadjuvant chemoradiotherapy

Supplementary Materials

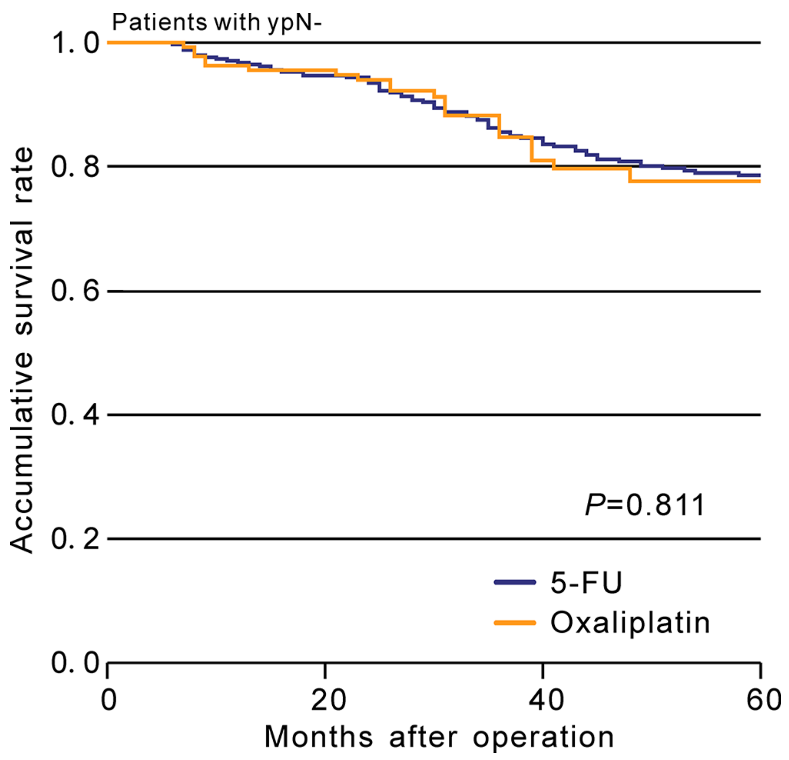

Supplementary Figure S1: Kaplan-meier comparison of overall survival among ypN- patients who received different postoperative treatment.

Supplementary Table S1: The difference of incidence of chemotherapy-related adverse events (Δ%) between oxaliplatin group and 5-FU group

	Total		yPN+		yPN-	
**Number of patients**						
Total	763		290		473	
Aged < 73	381		144		237	
Aged ≥ 73	382		146		236	
**Adverse event(Δ%)[a]**						
**Acute renal failure**						
Total	1.336%	*P* = 0.364	2.135%	*P* = 0.400	0.781%	*P* = 0.662
Aged < 73	−1.296%	*P* = 0.428	0.585%	*P* = 0.854	−2.548%	*P* = 0.150
Aged ≥ 73	5.355%	*P* = 0.035	4.438%	*P* = 0.275	5.963%	*P* = 0.068
**Infection**						
Total	0.742%	*P* = 0.731	1.766%	*P* = 0.543	0.262%	*P* = 0.930
Aged < 73	0.392%	*P* = 0.883	−0.753%	*P* = 0.858	1.131%	*P* = 0.743
Aged ≥ 73	2.054%	*P* = 0.558	4.438%	*P* = 0.275	0.875%	*P* = 0.863
**Neutropenia**						
Total	21.526%	*P* < 0.001	26.871%	*P* < 0.001	18.016%	*P* < 0.001
Aged < 73	26.104%	*P* < 0.001	32.191%	*P* < 0.001	22.333%	*P* < 0.001
Aged ≥ 73	15.653%	*P* < 0.001	21.423%	*P* = 0.002	11.579%	*P* = 0.019
**Nausea or vomiting**						
Total	16.712%	*P* < 0.001	16.278%	*P* = 0.001	17.028%	*P* < 0.001
Aged < 73	17.096%	*P* < 0.001	20.485%	*P* = 0.004	14.785%	*P* = 0.004
Aged ≥ 73	16.680%	*P* < 0.001	9.695%	*P* = 0.146	21.652%	*P* = 0.001
**Dehydration**						
Total	8.200%	*P* = 0.002	14.143%	*P* = 0.001	4.306%	*P* = 0.192
Aged < 73	7.430%	*P* = 0.033	13.294%	*P* = 0.030	3.583%	*P* = 0.387
Aged ≥ 73	9.883%	*P* = 0.016	15.150%	*P* = 0.018	6.431%	*P* = 0.225
**Diarrhea**						
Total	0.869%	*P* = 0.712	4.030%	*P* = 0.306	−1.231%	*P* = 0.675
Aged < 73	−0.593%	*P* = 0.856	5.602%	*P* = 0.317	−4.578%	*P* = 0.251
Aged ≥ 73	2.398%	*P* = 0.489	1.636%	*P* = 0.770	2.869%	*P* = 0.515
**Fever**						
Total	2.681%	*P* = 0.083	5.875%	*P* = 0.016	0.643%	*P* = 0.748
Aged < 73	5.568%	*P* = 0.008	7.441%	*P* = 0.046	4.339%	*P* = 0.080
Aged ≥ 73	−0.827%	*P* = 0.723	3.571%	*P* = 0.250	−3.643%	*P* = 0.265
**Anemia**						
Total	2.605%	*P* = 0.315	4.416%	*P* = 0.314	1.321%	*P* = 0.679
Aged < 73	3.724%	*P* = 0.342	5.686%	*P* = 0.408	2.261%	*P* = 0.630
Aged ≥ 73	−0.899%	*P* = 0.787	−0.149%	*P* = 0.977	−1.384%	*P* = 0.748
**Thrombocytopenia**						
Total	7.487%	*P* < 0.001	9.117%	*P* < 0.001	6.439%	*P* = 0.001
Aged < 73	5.568%	*P* = 0.008	3.595%	*P* = 0.258	6.839%	*P* = 0.013
Aged ≥ 73	9.958%	*P* < 0.001	16.216%	*P* < 0.001	5.759%	*P* = 0.028

**Delirium**						
Total	−0.993%	*P* = 0.588	1.638%	*P* = 0.534	−2.602%	*P* = 0.295
Aged < 73	−1.880%	*P* = 0.454	−0.753%	*P* = 0.858	−2.619%	*P* = 0.402
Aged ≥ 73	−0.004%	*P* = 0.999	3.571%	*P* = 0.250	−2.137%	*P* = 0.594
**IHD**						
Total	0.020%	*P* = 0.986	0.626%	*P* = 0.552	−0.260%	*P* = 0.884
Aged < 73	−0.849%	*P* = 0.488	−1.087%	*P* = 0.451	−0.661%	*P* = 0.709
Aged ≥ 73	1.646%	*P* = 0.432	2.703%	*P* = 0.085	1.160%	*P* = 0.723
**CHF**						
Total	−0.536%	*P* = 0.674	1.381%	*P* = 0.480	−1.752%	*P* = 0.294
Aged < 73	−0.091%	*P* = 0.946	−0.251%	*P* = 0.919	−0.024%	*P* = 0.988
Aged ≥ 73	−0.483%	*P* = 0.830	3.571%	*P* = 0.250	−3.093%	*P* = 0.322
**Cardiac dysrhythmia**						
Total	0.624%	*P* = 0.653	3.002%	*P* = 0.124	−0.850%	*P* = 0.655
Aged < 73	−0.137%	*P* = 0.933	1.672%	*P* = 0.558	−1.298%	*P* = 0.511
Aged ≥ 73	2.058%	*P* = 0.378	4.488%	*P* = 0.096	0.611%	*P* = 0.858

Abbreviation: CHF, congestive heart failure; IHD, ischemic heart disease.

[a]Δ% means the difference of incidence of chemotherapy-associated adverse events (Δ%) between oxaliplatin group and 5-FU group (Δ% = incidence of adverse events in oxaliplatin group minus incidence of adverse events in 5-FU group).
